# Progressive Cellular Architecture in Microscale Gas Chromatography for Broad Chemical Analyses

**DOI:** 10.3390/s21093089

**Published:** 2021-04-29

**Authors:** Weilin Liao, Xiangyu Zhao, Hsueh-Tsung Lu, Tsenguun Byambadorj, Yutao Qin, Yogesh B. Gianchandani

**Affiliations:** 1 Center for Wireless Integrated MicroSensing and Systems (WIMS2), University of Michigan, Ann Arbor, MI 48109, USA; liaoweil@umich.edu (W.L.); zxiangyu@umich.edu (X.Z.); leohtlu@umich.edu (H.-T.L.); tsenguun@umich.edu (T.B.); yutaoqin@umich.edu (Y.Q.); 2 Department of Electrical Engineering and Computer Science, University of Michigan, Ann Arbor, MI 48109, USA; 3 Department of Mechanical Engineering, University of Michigan, Ann Arbor, MI 48109, USA

**Keywords:** vapor, sampling, microvalve, volatile organic compound, phosphonate ester

## Abstract

Gas chromatography is widely used to identify and quantify volatile organic compounds for applications ranging from environmental monitoring to homeland security. We investigate a new architecture for microfabricated gas chromatography systems that can significantly improve the range, speed, and efficiency of such systems. By using a cellular approach, it performs a partial separation of analytes even as the sampling is being performed. The subsequent separation step is then rapidly performed within each cell. The cells, each of which contains a preconcentrator and separation column, are arranged in progression of retentiveness. While accommodating a wide range of analytes, this progressive cellular architecture (PCA) also provides a pathway to improving energy efficiency and lifetime by reducing the need for heating the separation columns. As a proof of concept, a three-cell subsystem (PCA3mv) has been built; it incorporates a number of microfabricated components, including preconcentrators, separation columns, valves, connectors, and a carrier gas filter. The preconcentrator and separation column of each cell are monolithically implemented as a single chip that has a footprint of 1.8 × 5.2 cm^2^. This subsystem also incorporates two manifold arrays of microfabricated valves, each of which has a footprint of 1.3 × 1.4 cm^2^. Operated together with a commercial flame ionization detector, the subsystem has been tested against polar and nonpolar analytes (including alkanes, alcohols, aromatics, and phosphonate esters) over a molecular weight range of 32–212 g/mol and a vapor pressure range of 0.005–231 mmHg. The separations require an average column temperature of 63–68 °C within a duration of 12 min, and provide separation resolutions >2 for any two homologues that differ by one methyl group.

## 1. Introduction

The detection and quantification of volatile organic compounds (VOCs) is critical in many applications, such as environmental monitoring, homeland security, and agriculture. Gas chromatographs (GCs) are frequently used for complex chemical analyses because they offer high selectivity, i.e., the ability to differentiate target analytes from interfering compounds. An essential element of GCs is a separation column, which is a fluidic channel coated with a stationary phase. Analytes interact with the stationary phase and elute from the column at characteristic times, which are measured by a common detector. Miniaturization of gas chromatographs is very attractive for portability and point-of-need field applications.

The concept of a microscale gas chromatograph (µGC) was first published in 1972 [1]. In 1979, Terry et al. reported the first µGC on a silicon wafer [2]. Since then, a variety of µGC systems and subsystems has been reported. The typical µGC architecture includes a preconcentrator or injector, a separation column, and a detector [3,4,5,6,7,8,9,10,11,12,13,14,15,16,17,18]. Some architectures have incorporated commercial off-the-shelf (COTS) valves to route the gas flow [3,4,5,6,7,8,9,10,11,12,13,14,15], whereas others have eliminated the need for valves by using bidirectional pumps [13,17].

In contrast to the typical architecture, which is considered one-dimensional, two-dimensional architectures can improve the separation of certain analytes by incorporating a second separation column with a different stationary phase [19]. In some cases, the first column contains a nonpolar stationary phase, whereas the second column contains a polar stationary phase. In “heart-cutting” architectures, only a fraction of the analytes eluting the first separation column is injected into the second separation column [19]. A recently reported two-dimensional GC used a first separation column to provide a rough separation and a second separation column that was tailored for one target analyte [20]. Comprehensive GC × GC uses a modulator to inject all the analytes eluting the first dimension column into one or more second dimension columns. A three-dimensional GC has also been reported [21]. Miniaturizations of these complex architectures have also been reported; these have incorporated selective combinations of microfabricated elements such as preconcentrators, injectors, separation columns, detectors, and modulators [22,23,24,25,26].

The temperature control and programming of the separation column is typically a major source of energy consumption in µGCs [12,27], especially those intended for a wide range of analytes. In general, high-volatility analytes benefit from a highly retentive separation column, which may incorporate a thick and retentive stationary phase or operate at a low temperature. Conversely, lower volatility analytes benefit from a less retentive separation column, which may incorporate a thinner stationary phase or operate at an elevated temperature. This represents a fundamental compromise.

**Figure 1:**
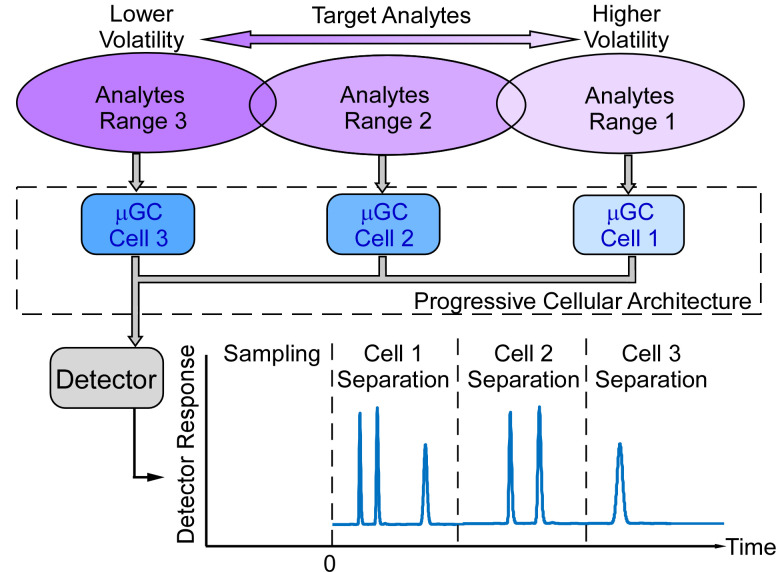
Progressive cellular architecture functional concept and the resulting chromatogram.

The progressive cellular architecture (PCA) reported in this work overcomes this compromise by incorporating a series of cells, each of which contains a preconcentrator and a separation column tailored to a specific volatility range of analytes (Figure 1). The outputs of the cells are routed to a common detector, which generates the resulting chromatogram. The cells are progressively cascaded in series to cover a broad range of vapors. In each cell, for the specific volatility range of analytes, the tailored separation column can rapidly and efficiently provide the desired separation resolution with modest temperature programming or none at all. Reduced heating requirements also extend the lifetime of the stationary phase coating within the separation columns, particularly when operating in complex environments and ambient air that may degrade these coatings at elevated temperatures [28]. This paper focuses on tailoring cells to the volatility range of targeted groups of analytes, although the polarity of the preconcentrator and separation column in each cell can also be tailored in principle.

The PCA uses the concept of multi-bed preconcentrators in an unconventional manner. Conventionally, multi-bed preconcentrators are packed with multiple sorbents arranged sequentially by adsorption strength [29,30]. During sampling, low-volatility analytes are trapped by the weak sorbent bed located upstream, whereas high-volatility analytes break through the weak bed and are trapped by the stronger sorbent bed located downstream. Although the different beds within a preconcentrator cause a partial separation during the sampling step, this preliminary separation is lost at the start of the separation cycle, when the entire preconcentrator is heated in order to create a single injection peak that is routed into the common separation column. Consequently, the partial separation achieved during sampling is lost in conventional systems. In contrast, within the PCA, each cell contains a single bed preconcentrator that targets a specific range of analytes; the preconcentrator of each cell is individually heated to inject these analytes into the corresponding separation column. Collectively, the sequential arrangement of cells achieves the function of a multi-bed preconcentrator but also preserves the separation that is naturally achieved during sampling (Figure 1).

There is an additional benefit to the preconcentrator arrangement used in the PCA. In conventional µGCs, the peaks of high-volatility analytes can be significantly broadened by slow injection, particularly if the separation column is not sufficiently retentive. In the PCA, because each column is tailored to a specific range of analytes, sufficient retention can be provided even for high-volatility analytes. This alleviates the requirement for rapid preconcentrator heating and ultrasharp analyte injection.

From an architectural perspective, the PCA is complementary to the µGC × µGC. The PCA is intended to provide energy efficient analysis for a wide volatility range of analytes, whereas the µGC × µGC is intended to enhance the separation of analytes with similar volatility values by adding a dimension of separation based on polarity. The separation columns in the PCA are arranged “in parallel” with each receiving a different volatility range of analytes. In contrast, the two-dimensional separation columns in the µGC × µGC are arranged “in series” with all columns receiving the entire range of analytes. The preconcentrator in each cell of the PCA only injects the analytes once. In contrast, the µGC × µGC uses a modulator to periodically inject the analytes eluting the first dimension column into the second dimension columns [22,24]. It is worth noting that the PCA can, in principle, be combined with µGC × µGC, as each PCA cell can selectively incorporate two-dimensional separation columns that are tailored for this cell.

Compared to other µGC architectures under limited energy budgets, the PCA accommodates a superior volatility range of analytes while maintaining moderate or superior separation (Table 1). Conventional one-dimensional µGC architectures accommodate a moderate volatility range of analytes while providing moderate separation performance, and are best suited for applications that have a narrow range of targets and simple interferences. Two-dimensional µGC architectures accommodate a moderate volatility range of analytes while providing superior separation performance, and are best suited for applications that have a narrow range of targets but complex interferences. In contrast, the PCA is amenable to applications with a broad range of target analytes.

In this work, a three-cell subsystem (PCA3mv) was built as a proof of concept for the PCA using a combination of microfabricated and commercial components. The PCA3mv was tested with various mixtures of both polar and nonpolar analytes. The theoretical rationale of the PCA is presented in Section 2; the design and implementation of the PCA3mv are described in Section 3; the experimental evaluation of the PCA3mv is described in Section 4, followed by the discussion and conclusion in Section 5.

## 2. Theoretical Rationale

In gas chromatography, two important metrics are the retention times of the analytes and the separation resolutions of target analyte pairs. The retention time (*t_Ri_*) of analyte *i* is the time taken to elute from a given column [31]:

(1) $$t_{Ri}=(1+k_{i})t_{0}$$

where *k_i_* is the retention factor for analyte *i*, and *t*_0_ is the elution time of an unretained analyte. The value of *k_i_* depends on the stationary phase material, its polarity, and its thickness; the temperature of the separation column; and the polarity and volatility of the analyte. In many applications, a large value of *k_i_* is undesirable because it increases *t_R_*, thereby forcing the carrier gas flow and column heating to be prolonged, consuming excessive energy.

The separation resolution (*R_s_*) of two analytes is also affected by the properties of the column and the analytes [31]:

(2) $$R_{s}=\frac{1}{4}{\left(N\right)}^{1/2}\left(\frac{\alpha -1}{\alpha }\right)\left(\frac{k_{2}}{1+k_{2}}\right);\;\alpha =\frac{k_{2}}{k_{1}}$$

where *α* is the separation factor, and *N* is the efficiency of the separation column, which is approximated as a constant for the given column geometry. The *α* is only dependent on the analyte properties, i.e., volatility and polarity. As evident from Equation (2), if *k*_2_ is <1, *R_s_* is highly sensitive to *k*_2_, decreasing rapidly with decreasing *k*_2_. If *k*_2_ is >10, an increase in *k*_2_ only contributes to a modest increase in *R_s_*. Considering Equations (1) and (2), to achieve large *R_s_* while maintaining small *t_R_*, *k_i_* in the range of 3–10 is favored for the target analytes. Note that Equations (1) and (2) are only applicable to isothermal separations; temperature programmed separations require a more complex analytical model.

The *R_s_* can be also determined experimentally from chromatograms [31]:

(3) $$R_{s}=\frac{1.18\Delta t_{R}}{PWHH_{1}+PWHH_{2}}$$

where Δ*t_R_* is the difference in retention time between two peaks; and the *PWHH*_1_ and *PWHH*_2_ are the peak widths at half height of the two peaks. Equation (3) assumes a perfectly Gaussian peak, whereas the actual peak shape may have peak distortion depending on the polarity of the analyte.

To evaluate the benefit of using a stationary phase thickness that is tailored to the target analytes, six hypothetical cases were analyzed; specifically, the retention time and separation resolution were estimated based on the carrier gas and flow, column type and length, and column temperature. In all cases, columns were assumed to have polydimethylsiloxane (PDMS) stationary phase, 3 m length and 250 µm inner diameter (ID). The carrier gas was assumed to be nitrogen with 0.6 sccm flow rate. The analytes were assumed to be alkanes C_3_–C_11_. (This type of analysis can be performed using any type of chromatogram modeler using well-established rules of solubility, including commercial modelers, e.g., Pro EZGC^®^ [32]). 

Cases 1–3 were used for comparing isothermal separations at 25 °C, which was assumed to be the ambient temperature. Case 1 incorporated a single column with 0.1 µm stationary phase thickness (Column B), and Case 2 incorporated a single column with 5.0 µm stationary phase thickness (Column A), whereas Case 3 mimicked a 2-cell PCA that incorporated Column A for alkanes C_3_–C_7_ and Column B for alkanes C_8_–C_11_. As evident from the computed results (Table 2), Case 1 required only 636 s analysis time for eluting C_11_, but provided *R_s_* ≤ 2.1 for the high-volatility analytes, C_3_–C_5_. Case 2 provided much higher *R_s_* ≥ 6 for C_3_–C_5_, but required 28,704 s analysis time, which was 45-fold longer than Case 1. Case 3 maintained *R_s_* ≥ 6 across alkanes C_3_–C_11_, and required 1309 s analysis time, which was only twice that of Case 1. For isothermal separation at the ambient temperature, the energy consumption resulted from the gas pump that provided the carrier gas flow, which was at a constant rate. Therefore, the energy consumption was proportional to the analysis time.

Broadening the analysis, Cases 4–6 were used to compare heated separations in which all the columns were temperature programmed to have a ramp rate of 30 °C/min starting at 25 °C at the beginning of the separations. Similar to Cases 1–3, Case 4 incorporated a Column B, Case 5 incorporated a Column A, whereas Case 6 mimicked a 2-cell PCA that incorporated Column A for alkanes C_3_–C_7_ and Column B for alkanes C_8_–C_11_. The computed retention times of various alkanes and separation resolution between pairs are shown in Table 3 for these cases. In order to assess the implications of these calculations, it is necessary to first estimate the total energy consumption.

The total energy required for heating a separation column (*E_total_*) is the sum of the energy needed to heat the thermal mass (*E_mass_*) and the heat dissipation (*E_diss_*) to the ambient by conduction and convection. The radiative thermal dissipation is negligible. Hence *E_diss_* can be expressed as:

(4) $$E_{diss}=\beta \cdot \Delta T_{avg}\cdot t_{a};\;\beta =K+h\cdot A$$

where *β* is the overall power consumption coefficient; *K* is the effective thermal conductance between the separation column and the ambient; *h* is the convection heat transfer coefficient; *A* is the effective device area available for convection; **Δ***T_avg_* is the average temperature difference between the separation column and the ambient during analysis; *t_a_* is the analysis time [33]. The *E_mass_* is proportional to maximum temperature difference between the separation column and the ambient (**Δ***T_max_*):

(5) $$E_{mass}=C_{th}\cdot \Delta T_{\max}$$

where *C_th_* is the thermal capacitance. The heat dissipation in microcolumns has been previously analyzed by Agah et al. [34]. Using graphs reported in that work, the values of *β* = 0.014 W/°C and *C_th_* = 0.7 J/°C can be assumed for the 3 m long silicon-glass microcolumn in this case study.

Table 4 shows the total analysis time, energy consumption, and column temperature for Cases 4–6. As evident from the computed results (Table 3 and Table 4), Case 4 required only 108 s analysis time for eluting C_11_ and an *E_total_* of 80.1 J, but *R_s_* **≤** 1.8 for C_3_–C_5_. Case 5 provided *R_s_* ≥ 4.9 for C_3_–C_5_, but required 278 s analysis time and an *E_total_* of 383.5 J, which were 2.5-fold and 4.8-fold of Case 4, respectively. Case 6 maintained *R_s_* **≥** 4.9 across C_3_–C_11_, and only required 231 s analysis time and an *E_total_* of 184.3 J. Compared to Case 5, Case 6 required only 83% analysis time, 35.6% *E_diss_*, 82.8% *E_mass_*, and 48.1% *E_total_*. This energy saving in Case 6 resulted from much lower *T_max_* and less analysis time than Case 5 even though Case 6 used two separation columns. To summarize, the 2-cell PCA in Case 3 and Case 6 improved overall analysis time and *E_total_* compared to a single column with a thick stationary phase, while maintaining higher *R_s_* across the analyte range compared to a single column with a thin stationary phase.

## 3. Design and Implementation

### 3.1. Subsystem Overview

**Figure 2:**
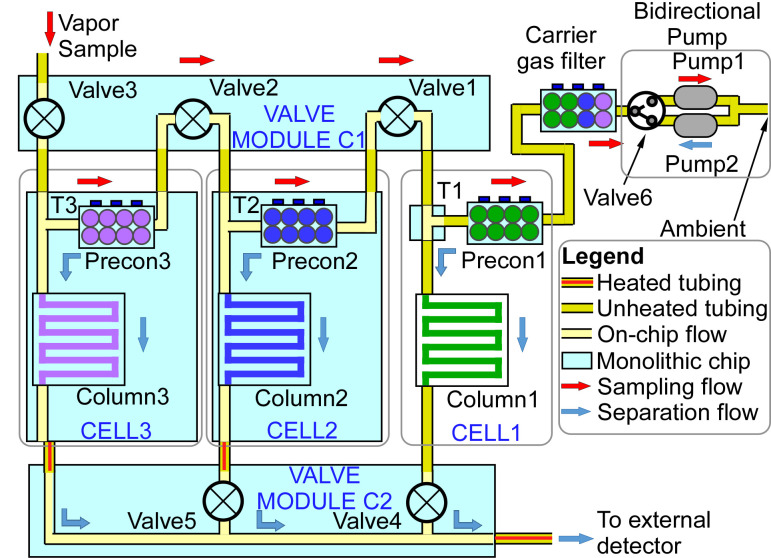
Structural arrangement of the three-cell (PCA3mv) subsystem.

The PCA3mv subsystem contained a number of elements. In particular, it incorporated three µGC cells as well as a carrier gas filter, two valve modules, a bidirectional pump module, and the controlling electronics (Figure 2). The µGC cells provided the preconcentration, injection, and separation of analytes. The valve modules and the pump module provided the sampling and separation flow. An external detector was used to evaluate the PCA3mv operation and performance.

Each cell incorporated a T-connector, a preconcentrator, and a separation column. To maximize the analyzable analyte range, the adsorption strength of the preconcentrator and the retentiveness of the separation column in each cell had to be significantly different from those of the other cells. Cell 1, which targeted the volatility range from C_3_ to C_5_, incorporated the strongest sorbent in its preconcentrator and the most retentive stationary phase in its separation column. Among the commercially available sorbents, Carboxen™ 1000 (#10477-U, Sigma Aldrich, St. Louis, MO, USA) has the highest surface area of 1200 m^2^/g, hence it was selected for the Cell 1 preconcentrator. Similarly, among the common stationary phases, polydivinylbenzene (pDVB) is a non-polar, porous, and highly retentive material, hence it was selected for the Cell 1 separation column. Cell 2, which targeted C_5_ to C_8_, incorporated a moderate sorbent and a less-retentive stationary phase. Carbopack™ X (#10437-U, Sigma Aldrich, MO, USA) has a surface area of 240 m^2^/g, which is typically used for this volatility range; hence, it was selected for the Cell 2 preconcentrator. A relatively thick layer of PDMS (5 µm) was selected for the Cell 2 separation column. Cell 3, which targeted C_8_ to C_15_, incorporated the weakest sorbent and least-retentive stationary phase. Carbograph™ 2 (#1728, Grace Davison Discovery Science, IL, USA) has a surface area of 10 m^2^/g; hence, it was selected for the Cell 3 preconcentrator. A relatively thin layer of PDMS (0.3 µm) was selected for the Cell 3 separation column.

The gas flow was controlled by two valve modules; Valve Module C1 incorporated three 2-way valves (Valve1, Valve2, and Valve3) and Valve Module C2 incorporated two 2-way valves (Valve4 and Valve5). Valve Module C1 was connected to the preconcentrators, whereas Valve Module C2 was connected to the separation columns. The gas flow was provided by a bi-directional pump module, which included Pump1 for providing sampling flow, Pump2 for providing separation flow, and a 3-way valve (Valve6) for selecting between these two options. A stand-alone microfabricated carrier gas filter was connected between Precon1 and Valve6 to capture chemicals and moisture that may be present in the carrier gas used in separation.

During vapor sampling, Pump1 was actuated, whereas the valves were switched to allow gas flow through the preconcentrators while blocking the flow through the separation columns. The preconcentrators were connected in series, allowing the flow to pass sequentially from the weakest Precon3 to the strongest Precon1. The upstream preconcentrator, Precon3, trapped the analytes with the lowest volatility while allowing more volatile analytes to break through to Precon2 and Precon1. Therefore, during vapor collection, a *de facto* preliminary separation was achieved. In this work, the preliminary separation was performed on the basis of analyte volatility, as the preconcentrators only incorporated nonpolar sorbents. If the preconcentrators incorporated polar sorbents, the preliminary separation would also depend on the analyte polarity.

At the conclusion of the sampling step, the separation step was initiated. The carrier gas used for separation was ambient-air scrubbed by a carrier gas filter. During separation, Pump2 was actuated, and the valves were switched to direct the carrier gas sequentially into Cell 1, Cell 2, and Cell 3. In each cell, the preconcentrator thermally desorbed the collected analyte molecules and from there these were transported to the corresponding separation column. The eluents from all the cells were routed through a single outlet toward the external FID.

### 3.2. Microfabricated Components

**Figure 3:**
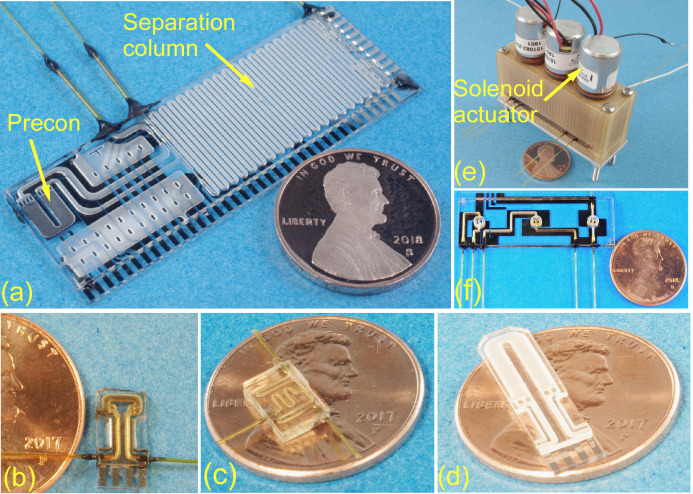
Microfabricated components within the PCA3mv: (**a**) monolithic chip, (**b**) preconcentrator, (**c**) T-connector, (**d**) carrier gas filter (**e**) Valve Module C1, and (**f**) valve module chip.

A variety of µGC components have been reported, typically as standalone elements: preconcentrators [35], valves [36,37], separation columns [38,39,40], detectors [41,42,43,44,45,46], and pumps [47,48,49]. In the PCA3mv, Cell 2 and Cell 3 each used a monolithic chip that integrated a preconcentrator, a separation column, and a T-connector (Figure 3a); Cell 1 did not use a monolithic chip as the coating method for the Column1 stationary phase has not been developed. The microfabricated Column2 and Column3 were designed as serpentine channels with 0.6 m total length, 500 µm width, and 160–180 µm height. As noted previously, Column2 incorporated a 5.0 µm thick PDMS stationary phase, whereas Column3 incorporated a 0.3 µm thick PDMS stationary phase. Precon2 and Precon3 were designed as 3.5 µL U-shaped chambers for accommodating sorbent particles. Each chamber incorporated two arrays of micropillars for confining the sorbent particles and a loading port for loading the sorbent particles. Precon2 was packed with an estimated 1.4 mg of Carbopack™ X. Precon3 was packed with an estimated 2.4 mg of Carbograph™ 2.

The Cell 2 monolithic chip, the Cell 3 monolithic chip, and the carrier gas filter each consisted of two glass dies bonded together. The top glass die included gas flow channels created by sandblasting (provided by Bullen Ultrasonics Inc., Eaton, OH, USA, and Ikonics^®^ Corporation, Duluth, MN, USA). The bottom glass die included thin-film metal heaters and thermistors to allow servo-controlled heating. The die bonding used an epoxy (#377, Epoxy Technology, Billerica, MA, USA). The packed sorbent masses were estimated based on the sorbent chamber volumes and the sorbent densities. After sorbent packing, the loading ports were sealed with epoxy.

Precon1 was a standalone preconcentrator (Figure 3b) with an inner volume of 2.4 µL, and was packed with an estimated 1.2 mg of Carboxen™ 1000. The connector T1 (Figure 3c) was a stand-alone microfabricated T-connector for connecting Precon1, Valve1, and Column1. The carrier gas filter (Figure 3d) was designed as a chamber with an inner volume of 12.5 µL and was packed with an estimated 2.1 mg of Carboxen™ 1000, 1.7 mg of Carbopack™ X, and 2.9 mg of Carbograph™ 2. Precon1, T1, and the carrier gas filter were also microfabricated and assembled in a manner similar to Cell 2 and Cell 3 monolithic chips.

The microvalves contained in Valve Module C1 and Valve Module C2 included gas flow channels, valve seats, and embedded thin-film metal heaters and thermistors (Figure 3e,f). These were monolithically fabricated and assembled in a manner similar to Cell 2 and Cell 3 monolithic chips as well. Each microvalve incorporated a prefabricated polyimide valve membrane which was actuated by a latching solenoid actuator (#151082-234, Ledex, CA, USA). Each valve module was housed within a custom housing 3D-printed from Ultem 1010 [50].

All the microfabricated chips were supported by glass spacers and mounted on a common printed circuit board (PCB). The spacers were 1 mm thick and custom-sized to support certain portions of these chips while cantilevering the sorbent chambers in the preconcentrators and the carrier gas filter above the PCB. Cantilevering provided thermal isolation of preconcentrators and the carrier gas filter from the PCB.

The microfabricated chips were fluidically interconnected with 250 µm ID guard columns (#10010, Restek, PA, USA), which were uncoated capillary tubes with surface treatment to minimize adsorption. To prevent surface adsorption of highly adsorptive analytes in the guard columns, resistive heating was provided by externally wrapped copper wires. The temperature of the guard columns was monitored by a commercial thermistor (#490-4801-1-ND, Digi-key Electronics, Thief River Falls, MN, USA).

### 3.3. Other Components

Column1 was a COTS porous layer open tubular (PLOT) column of 1 m length, 250 µm ID, and with 8 µm thick polydivinylbenzene stationary phase (#19764, Restek, PA, USA). Resistive heating was provided by an externally wrapped nichrome wire. The temperature was monitored by a commercial thermistor (#490-4801-1-ND, Digi-key Electronics, MN, USA). Column1 was placed within a custom housing 3D-printed from Ultem 1010. (After a method has been developed for coating polydivinylbenzene or an equivalent stationary phase in microfabricated columns, the COTS Column1 can be replaced with a microfabricated version.)

Pump1 was a miniature diaphragm pump (#20KD, Boxer, Ottobeuren, Germany) which provided a relatively high flow rate (up to 10 sccm) for fast sampling. Pump2 was a miniature diaphragm pump (#NMP03KPDC-L, KNF Neuberger, Inc., Trenton, NJ, USA) which provided a moderate flow rate (0.5–1 sccm) for effective separation. Valve6 was a 3-way PTFE isolation valve (#NR1O-3-12, Clippard, OH, USA).

### 3.4. Electronic Interface

**Figure 4:**
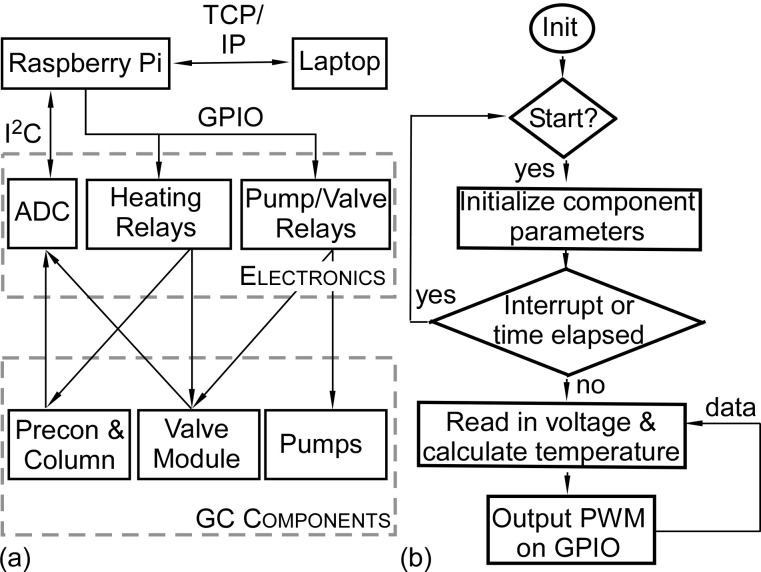
(**a**) Electrical connections between the microcontroller, the laptop with the user interface, the integrated circuits, and the GC components. (**b**) The flow chart of the control software illustrating closed-loop temperature control of the components.

The fluidic components were electrically interfaced with a microcontroller (#Raspberry Pi 3, Raspberry Pi Foundation, Cambridge, UK) through a dedicated PCB, which included analog-to-digital converters (ADCs), relays, and other power electronics (Figure 4a). The microcontroller communicated with ADCs via inter-integrated circuit (I^2^C) protocols for temperature sensing and used general-purpose input and output (GPIO) pins to control the relays for component heating and the actuation of valves and pumps. Heating profiles for the components were set in the user interface and controlled by a proportional integral derivative control loop (Figure 4b). The sampling flow and separation flow were controlled by switching the pumps and the three-way valve. The microcontroller used a standard transmission control protocol and Internet protocol (TCP/IP) to communicate with a laptop equipped with a customized graphical user interface, allowing remote control of the PCA3mv. 

**Figure 5:**
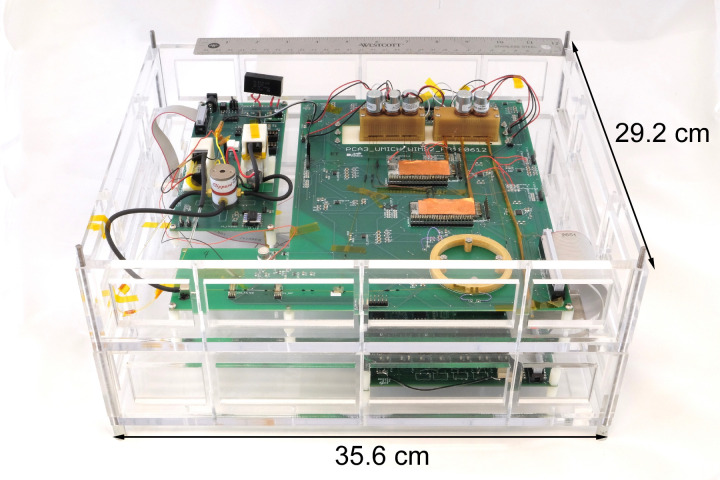
Photograph of the PCA3mv implementation. The PCA3mv subsystem has a footprint of 29.2 cm × 35.6 cm.

To operate the PCA3mv, the user preset the target temperature and timing of each component in the user interface. After initialization, the microcontroller provided closed-loop control at a loop frequency of 10 Hz. All components were housed within a clear cast acrylic box 35.6 × 29.2 × 15.2 cm^3^ (Figure 5). The overall PCA3mv had displaced volume of 0.6 L.

## 4. Experimental Evaluation

Prior to evaluating the PCA3mv, the microfabricated separation columns were individually evaluated using a benchtop GC. After that, all the components were assembled into the PCA3mv subsystem, which was tested for the sampling and separation of various analytes at different concentrations and humidity levels. The PCA3mv was also tested for sample carryover from run to run. In principle, a variety of microfabricated detectors, such as capacitive, photoionization, and surface acoustic wave detectors can be used within the PCA3mv [41,42,43,44,45,46]. However, in this work, a commercial flame ionization detector (FID) was used to evaluate the preconcentration and separation. The FID does not broaden any peaks in a substantive way, and hence provides truer representation for the performance of the PCA3mv [31].

**Figure 6:**
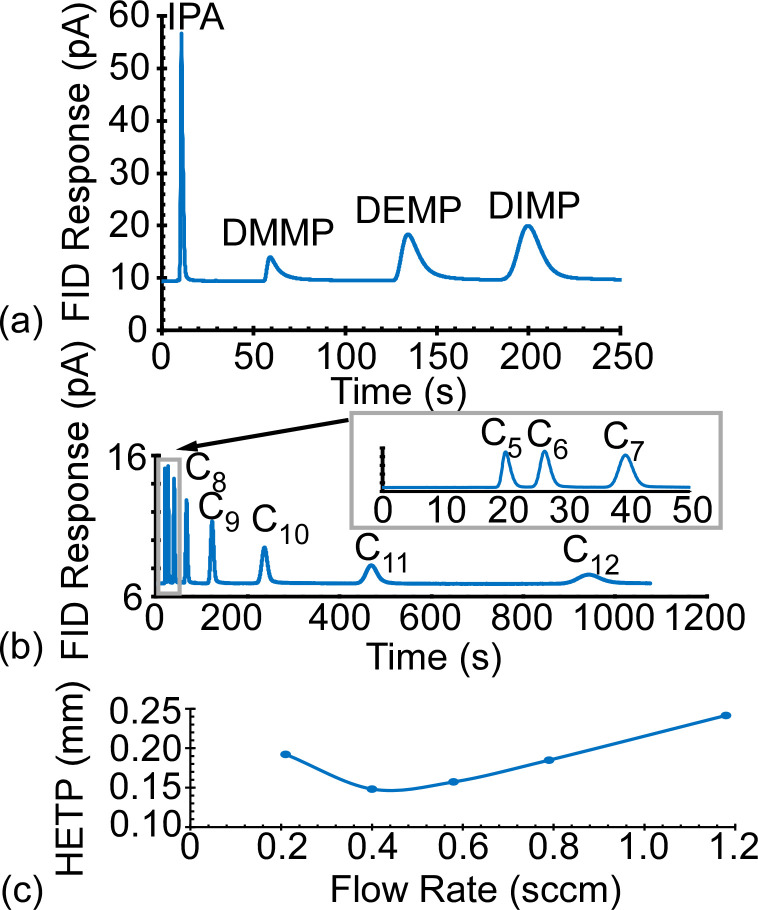
Chromatograms of the separation of (**a**) IPA and phosphonate esters, and (**b**) alkanes C_5_–C_12_ in Column2. (**c**) The *HETP* of Column2 calculated using C_10_ at 80 °C at 0.2–1.2 sccm. Column3 showed similar *HETP* results.

Prior to conducting full functional testing, the performance of the microfabricated separation columns within the Cell 2 and Cell 3 monolithic chips was evaluated by connecting the separation column between the inlet and the FID of a benchtop GC (#Agilent 7890, Agilent, CA, USA). For this evaluation, the flow path through the on-chip preconcentrator was temporarily blocked by an external septum. Pre-mixed analytes in liquid form were injected and vaporized at the inlet of the benchtop GC using splitless injection. The analytes were routed into the chip at on-chip T-connector, i.e., T2 for Cell 2 and T3 for Cell 3 (Figure 2). The eluents from the separation column were measured by the FID. A typical Column2 separation of a mixture of isopropyl alcohol (IPA) and phosphonate esters is shown in Figure 6a, whereas that of a mixture of alkanes from C_5_ to C_12_ is shown in Figure 6b. Operated at 80 °C and a 0.8 sccm N_2_ flow rate, the IPA and phosphonate esters were separated within 220 s, whereas the alkanes were separated within 1000 s. 

The height equivalent to a theoretical plate (*HETP*) and the theoretical plate number (*N*) are measures of separation column efficiency that can be extracted from the chromatogram as follows [31]:

(6) $$N=5.54{\left(\frac{t_{R}-t_{0}}{PWHH}\right)}^{2}$$

(7) $$HETP=\frac{L}{N}$$

where *L* is the length of the column. Higher *N* and lower *HETP* indicate higher column efficiency. The *HETP* of the microfabricated Column2 in this work reached a minimum of 0.14 mm at 0.4–0.5 sccm flow rate, as calculated using the C_10_ peak with a measured *t_R_* = 236.5 s, a calculated *t*_0_ = 13.4 s, and a measured *PWHH* = 8.26 s (Figure 6c). The value of *t*_0_ was calculated as the time for an unretained analyte to travel through the separation column and the guard column connections at the tested flow rate. The *HETP* value corresponded to 4048 theoretical plates and 6747 plates/m. These values are comparable to other reported microfabricated columns [51,52]. Column3 provided similar performance for lower volatility analytes.

To test the fully assembled PCA3mv, analytes were pre-mixed in liquid form, and injected into a first 2 L dilution bottle to form a relatively high-concentration vapor. The high-concentration vapor was subsequently diluted into lower concentrations by using a gas tight syringe to transfer an aliquot of the vapor into a second dilution bottle. Two guard columns were connected to the second dilution bottle: one was used for connecting to the PCA3mv, whereas the other vented to the ambient air to prevent a vacuum in the bottle during sampling. While this setup was not able to provide precise concentrations in a sustained manner, it was appropriate for a preliminary evaluation. During sampling, the dilution bottle was connected to the sample inlet of the PCA3mv, whereas the outlet of the PCA3mv was temporarily blocked to prevent the entrance of the ambient air through Column3. During separation, the outlet of the PCA3mv was connected to an FID of the benchtop GC.

Each analytical run of the PCA3mv consisted of three steps: purging, sampling, and separation. The purging step was used to remove any contaminants within the preconcentrators remaining from the preceding run. During purging, Pump2 was actuated, whereas Valve1, Valve2, and Valve3 were switched to connect all the preconcentrators in series. Then the preconcentrators were heated to 150–200 °C for 30 s to desorb the residue, which was purged out of the sample inlet. At the beginning of sampling, the carrier gas filter heated by a 58 s, 150 °C thermal pulse, which removed any ambient contaminants trapped in the filter during the preceding run and regenerated the filter for the ongoing run. The PCA3mv was operated to collect the vapor sample for 30 min using ≈5 sccm flow rate.

**Figure 7:**
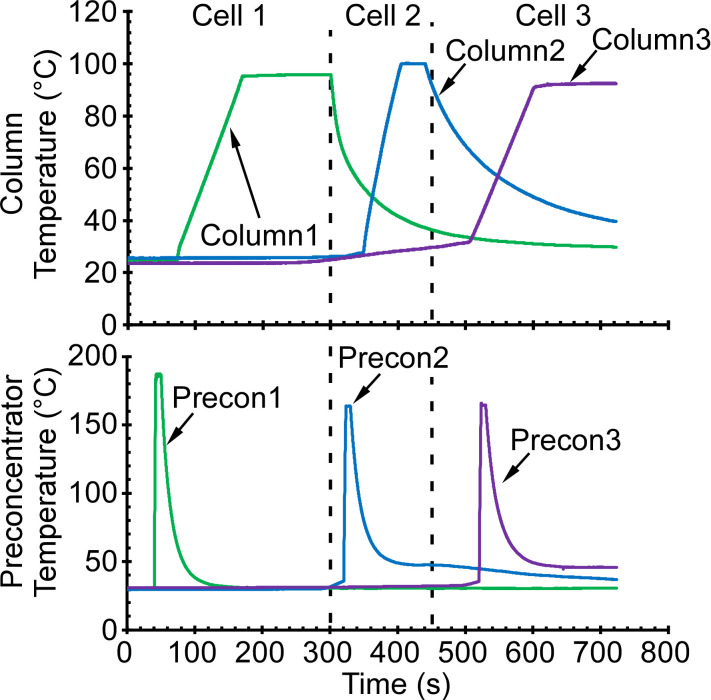
Temperature control for the preconcentrators and the separation columns.

For the separation in each cell, the air flow rate was maintained at ≈0.9 sccm. The preconcentrator was heated from room temperature to 170–180 °C in 2 s and maintained for 8 s to perform desorption (Figure 7). Column1 provided temperature programmed separation ramping from room temperature to 100 °C using ≈0.5 °C/s ramp rate. Column2 provided temperature programmed separation ramping from room temperature to 100 °C using ≈1.3 °C/s ramp rate. Column3 provided temperature programmed separation ramping from room temperature to 100 °C using ≈0.6 °C/s ramp rate. Valve Module C2 was maintained at 80 °C during Cell 2 and Cell 3 separation. The guard columns in the separation paths of Cell 2 and Cell 3 were maintained at 60–70 °C. The guard column of Cell 1 was maintained at room temperature, because the analytes in Cell 1 were sufficiently volatile.

**Figure 8:**
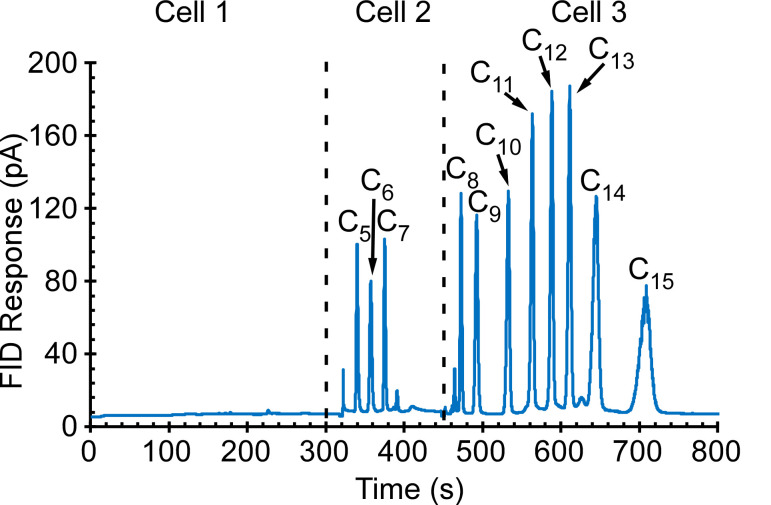
The PCA3mv chromatogram of 30 min sampling of 100 ppb of alkanes C_5_–C_15_. Alkanes C_5_–C_7_ were separated in Cell 2, whereas alkanes C_8_–C_15_ were separated in Cell 3.

**Figure 9:**
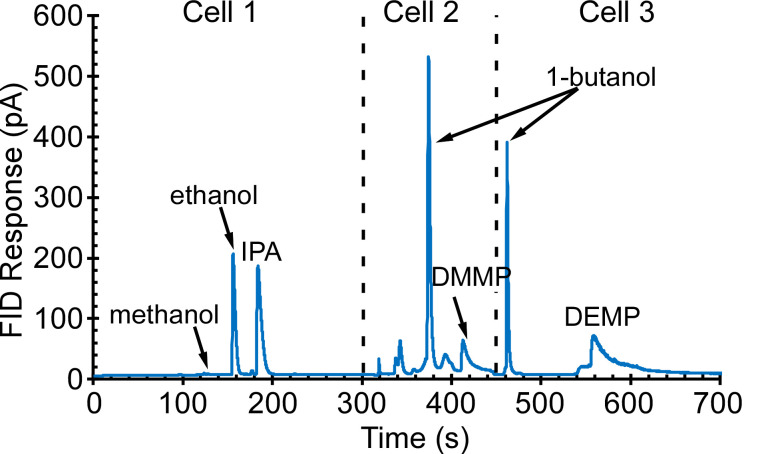
The PCA3mv chromatogram of 30 min sampling of 1 ppm of alcohols and phosphonate esters. Methanol, ethanol, and IPA were separated in Cell 1, 1-butanol and DMMP were separated in Cell 2, and 1-butanol and DEMP were separated in Cell 3.

**Figure 10:**
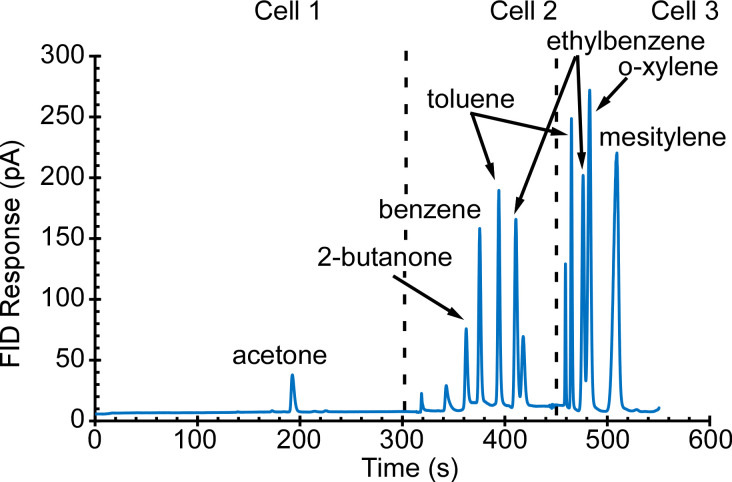
PCA3mv chromatogram of 30 min sampling of 200 ppb of aromatics. Acetone was separated in Cell 1, 2-butanone, benzene, toluene, and ethylbenzene were separated in Cell 2, and ethylbenzene, o-xylene, and mesitylene were separated in Cell 3.

Initial tests were directed at the collection and separation of alkanes C_5_–C_15_ with a concentration of 100 ppb; typical results are presented in Figure 8. The alkanes C_5_–C_7_ were retained and separated in Cell 2, and alkanes C_8_–C_15_ were separated in Cell 3. Other tests were directed at alcohols, in particular, methanol, ethanol, IPA, and butanol; and phosphonate esters, in particular, dimethyl methylphosphonate (DMMP) and diethyl methylphosphonate (DEMP)—all at a concentration of 1 ppm. Typical results are presented in Figure 9. Methanol, ethanol, and IPA were separated in Cell 1; 1-butanol and DMMP were separated in Cell 2; 1-butanol and DEMP were separated in Cell 3. Yet other tests were directed at ketones (acetone and butanone) and aromatics (benzene, toluene, ethylbenzene, o-xylene, and mesitylene)—all at a concentration of 200 ppb; typical results are presented in Figure 10. Acetone was separated in Cell 1; 2-butanone, benzene, toluene, and ethylbenzene were separated in Cell 2; toluene, ethylbenzene, o-xylene, and mesitylene were separated in Cell 3. The volatility of each of the tested analytes is represented by its Kovats retention index (RI), which is an indicator of the analyte retention time relative to *n*-alkanes [31]. The RI for the tested analytes ranged from 370 to 1500. 

The PCA3mv provided *R_s_* ≈ 4.7 for the first two eluting analytes (methanol and ethanol) with a RI of 370 and 443, respectively. The PCA3mv also provided *R_s_* ≈ 2.3 for the two analytes with lowest vapor pressure (C_14_ and C_15_) with a RI of 1400 and 1500, respectively. All the separations were performed with Δ*T_avg_* of 41.7 °C over 12 min using analytes with molecular weights ranging from 32–212 g/mol and vapor pressures ranging from 0.005–231 mmHg.

Phosphonate esters are typically used as simulants for chemical warfare agents due to similarities in their molecular structures and chemical properties [53]. The high polarity of the phosphonate esters results in their strong adsorption on surfaces along the flow path and can manifest as the absence of a detectable peak or other atypical response [54]. In tests performed with the PCA3mv, the phosphonate esters were successfully separated and detected. As expected, however, the retention behaviors were different from the nonpolar analytes. The vapor pressure of DMMP is 0.96 mmHg, which is lower than those of octane, nonane, and decane (14.05 mmHg, 4.45 mmHg, and 1.43 mmHg, respectively). However, the DMMP peak appeared in Cell 2, whereas the octane, nonane, and decane peaks appeared in Cell 3. This indicated that DMMP broke through the Carbograph™ 2 sorbent in Precon3 more easily than nonpolar analytes of comparable vapor pressure. The vapor pressure of DEMP is 0.01 mmHg, similar to that of tetradecane. In the experimental results (Figure 9), DEMP eluted in Cell 3 with a retention time similar to undecane, which was slower than expected. In addition, the DEMP peak presented a large tail. These effects were likely caused by the high adsorption of the phosphonate ester on the surface of fluidic channels with insufficient deactivation.

**Figure 11:**
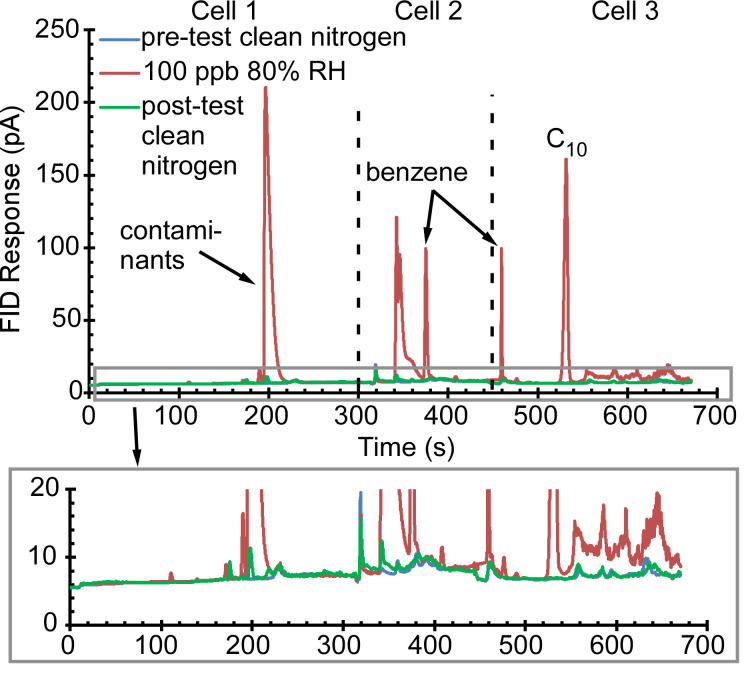
The PCA3mv chromatogram of the bracket test with 3 consecutive analytical cycles. Cycle: clean nitrogen. Cycle 2: IPA, benzene, C_10_ at 80% relative humidity. Cycle 3: clean nitrogen. Cycle 3 showed almost the same chromatogram as Cycle 1, indicating minimal sample carryover.

Bracket tests comprised of three consecutive cycles were intended to show sample carryover from run to run. These included a clean nitrogen sample in the first cycle, then a mixture of IPA, benzene, and C_10_ in the second, and again a clean nitrogen sample in the third. As evident in Figure 11, the chromatogram from the third cycle matched that of the first, indicating that the IPA, benzene, and C_10_ collected in the second cycle were removed from the PCA3mv with minimal sample carryover from run to run. In the second cycle, the benzene peak in Cell 2 had a height of 94 pA, whereas in the first and third cycles, the peak heights at the retention time of benzene in Cell 2 were 3 pA and 3.6 pA respectively. The benzene peak in Cell 2 had ≈4% carryover.

**Figure 12:**
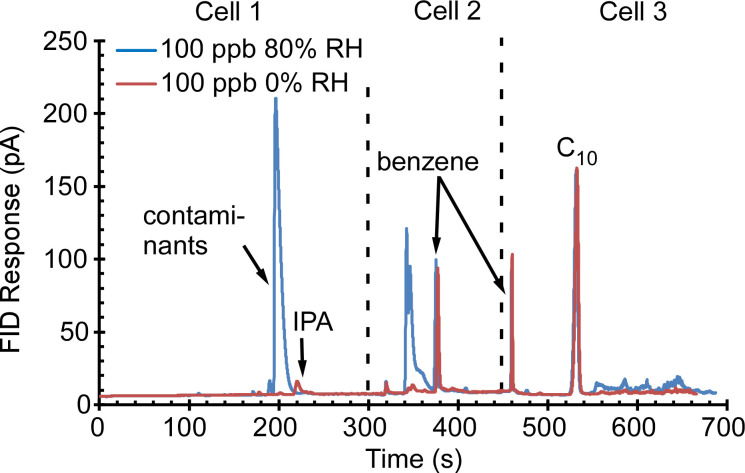
The PCA3mv chromatogram of IPA, benzene, and decane at 80% relative humidity and 0% relative humidity. The benzene and decane peaks in Cell 2 and Cell 3 showed minimal humidity impact.

The impact of humidity was evaluated using mixtures of IPA, benzene, C_10_ (each 100 ppb) in air with 0 and 80% relative humidity (RH). Based on a humidity calculator [55], the sample with 80% RH was prepared by injecting 30.5 µL of high-purity, double-distilled water (#WX0003-6, Sigma Aldrich, MO, USA) into the 2 L dilution bottle and then allowing the water enough time to vaporize and reach equilibrium before sampling. The VOCs used in this set were selected to allow Cell 1 to be evaluated with IPA, Cell 2 to be evaluated with benzene, and Cell 3 to be evaluated with C_10_. The resulting chromatograms (Figure 12) showed that the retention peaks of benzene and C_10_ were not affected by the humidity: the measured peak areas of benzene and C_10_ showed <2% difference between the chromatograms at 0 and 80% RH. In principle, the FID is not responsive to water. However, in the run at 80% RH, two additional peaks were observed in Cell 1 and Cell 2, likely resulting from possible contaminants in the water that was used for humidity. Also at 80% RH, the IPA peak disappeared from the Cell 1 chromatogram segment; this observation is discussed in Section 5.

## 5. Discussion and Conclusions

Overall, the experimental results show that PCA3mv effectively separated analytes with good resolution (*R_s_* > 2.3) over a wide range of volatility covering RI 370–1500. Compared to previously reported representative µGCs (such as [11,12,17,23,24]), the PCA3mv results covered analytes with a larger span of retention index while maintaining high separation resolution over the entire span. Further, the PCA3mv separations only required an average column temperature of 42 °C and a duration of 12 min, both of which are smaller than those reported for other µGC architectures [11,23,24] underscoring the potential for lower energy consumption. Among the representative one-dimensional µGCs, Zhou [11] reported analytes over an RI range of 590–1183, using an average column temperature of 70 °C and a duration of 15 min. Wang [12] reported an RI range of 370–1200, using 65 °C and only 3 min, but in the RI range of 370–500 the separation was less effective (with *R_S_* < 1). Qin [17] reported separations performed at room temperature within 10 min, but the analyzed range was smaller (with 500 < RI < 1000) and the *R_S_* was modest (≈1) for the RI range of 500–600. The representative two-dimensional µGCs [23,24] provided adequate separation (with *R_S_* ≥ 1.4 for most pairs of tested analytes) over relatively small RI ranges (524–831 in [23] and 600–1200 in [24]), while requiring higher average column temperatures of 50–80 °C and longer separation durations of 14–17 min.

The experimental results of the PCA3mv also showed in humidity tests that Cell 2 and Cell 3 were minimally affected; this was expected because of the lower water sorption of the sorbents used in Precon2 and Precon3 [56]. At 80% RH (Figure 12), the disappearance of the IPA peak in Cell 1 was likely caused by the adsorption of water molecules by the hydrophilic sorbent Carboxen™ 1000 in Precon1 [57]. This can be potentially addressed by using a hydrophobic sorbent (e.g., Carboxen™ 1003).

A characteristic of note for highly adsorptive analytes such as DMMP and DEMP was the peak tailing, where the trailing fraction of the peak was significantly wider than the leading fraction, especially at a height close to the baseline (Figure 9). This can be attributed to surface adsorption in the flow path. It can be reduced in the future by improving the heating uniformity and surface deactivation of the flow path. It can also be reduced by further monolithic integration, which would reduce the fluidic connections.

Some analytes such as 1-butanol, toluene, ethylbenzene, benzene, and o-xylene, appeared in two neighboring cells. For these analytes, a primary cell could be identified as the cell that provided sufficient retention of the analytes or dominated the fractional distribution of the analyte molecules. For the more volatile 1-butanol, benzene, and toluene, the peaks in Cell 3 appeared early (within the first 40 s) without proper retention; hence, these peaks should simply be ignored in favor of the corresponding peaks in Cell 2, which represented sufficient retention. For the less volatile ethylbenzene and o-xylene, Cell 3 provided proper separation that was similar to Cell 2 but with larger peak heights; hence, Cell 3 should be used as the primary cell. To the extent that the distribution of the analytes between two cells was deterministic over certain operating conditions, quantitative analysis of these analytes can be provided by calibrating the primary cell for the fractional distribution in addition to the peak heights. For the construction of a chemical library, retention parameters (i.e., retention times at prescribed temperature and flow rate) can be extracted from the primary cell, similar to the process used for conventional one-dimensional architectures. Therefore, although such cross-cell distributions may be a disadvantage, their negative impact is limited and controllable. This topic requires further investigation.

In conclusion, this work showed that by cascading heterogeneous µGC cells, the PCA provides partial separation during sampling and further separation of the targeted group of analytes in each cell. As a proof of concept, the PCA3mv was built and tested with polar and nonpolar analytes having molecular weights of 32–212 g/mol. The PCA3mv showed that the separations can be completed within 12 min with *R_s_ >* 2 for any pair of tested homologues that differed by one methyl group. Preliminary tests showed encouraging results about the immunity of the subsystem to analyte carryover and to humidity in the sample. Future work includes characterization of the response to varying analyte concentrations, and characterization of the distribution of analytes between cells. Additional improvements can be envisioned for PCA. Cells can be added to further increase the range of targeted analytes. If the application specifically requires separating compounds with similar volatility but different polarity, polar separation columns can be utilized. Architectural variations can be envisioned for the PCA, such as replacing Valve1 and Valve2 with another valve downstream of Column3 to decrease the number of valves needed. In addition, microfabricated detectors can be incorporated upstream of the Valve Module C2. Microfabricated bidirectional pumps and detectors can be implemented to provide a completely microfabricated analytical system. A higher level of monolithic integration can be implemented to provide a more compact system while also eliminating the need for heating the guard-column connections. By increasing the compactness of the system, field deployment of the system can be envisioned in a variety of spaces such as industrial plants and urban settings. Other possible applications include online sampling and separation systems in complex environments, concentration enrichment in low concentration environments, and solvent elimination from multiple component mixtures.

## Figures and Tables

**Table 1:** General comparison of µGC architectures under limited energy budgets.

Architecture	Volatility Range	Separation	Application Environments
Conventional (one-dim.)	Moderate	Moderate	Narrow targets, simple interference
Heart-cutting (two-dim.)	Moderate	Superior	Narrow targets, complex interference
Comp. GC × GC (two-dim.)	Moderate	Superior	Narrow targets, complex interference
PCA	Superior	Moderate or Superior	Broad targets, simple or complex interference

**Table 2:** Computed ^a^ values of retention times of various alkanes and separation resolution between pairs of alkanes at 25 °C for Cases 1–3.

	Case 1		Case 2		Case 3	
Stationary Phase Thickness (µm)	Col. B Only: 0.1		Col. A Only: 5.0		Col. A: 5.0 Col. B: 0.1	
Total Analysis Time (s)	636		28,704		A: 675/B: 634 Overall: 1309	
	*t_R_* (s)	*R_s_*	*t_R_* (s)	*R_s_*	*t_R_* (s) ^b^	*R_s_*
Propane	15.3		30.0		30.0	
		0.7 ^c^		6.0		6.0
Butane	15.7		46.2		46.2	
		2.1		12.8		12.8
Pentane	16.8		93.9		93.0	
		6.6		24.0		24.0
Hexane	19.8		242.4		242.4	
		15.6		33.8		33.8
Heptane	29.4		675.0		675.0	
		25.5		27.0		33.7
Octane	53.4		1787		53.4	
		33.7		38.2		33.7
Nonane	115.8		4694		115.8	
		35.2		35.9		35.2
Decane	270.6		11,868		270.6	
		35.2		35.9		35.2
Undecane	636.0		28,704		633.6	
		

^a^ All cases computed the separation of alkanes C_3_–C_11_ using a 3 m long, 250 µm ID separation column. ^b^ In Case 3, Col. A separated propane–heptane, whereas Col. B separated octane–undecane. ^c^ Red font indicates undesirable outcomes compared to other cases.

**Table 3:** Computed ^a^ values of retention time of various alkanes and separation resolution between pairs of alkanes for temperature programmed Cases 4–6.

	Case 4		Case 5		Case 6	
Stationary Phase Thickness (µm)	Col. B Only: 0.1		Col. A Only: 5.0		Col. A: 5.0 Col. B: 0.1	
	*t_R_* (s)	*R_s_*	*t_R_* (s)	*R_s_*	*t_R_* (s) ^b^	*R_s_*
Propane	15		26.4		26.4	
		0.6 ^c^		4.9		4.9
Butane	15.6		36		36	
		1.8		8.2		8.2
Pentane	16.2		56.4		56.4	
		5.4		11.5		11.5
Hexane	18.6		90.6		90.6	
		11.9		11.7		11.8
Heptane	25.2		129		129	
		17.8		11.4		21
Octane	37.2		167.4		37.2	
		20.5		10.7		20.5
Nonane	57		206.4		57	
		18.6		9.4		18.6
Decane	81.6		243.6		81.6	
		18.6		9.4		18.6
Undecane	108		277.8		108	
		

^a^ All cases computed the separation of alkanes C_3_–C_11_ using a 3 m long, 250 µm ID separation column. All cases used a 30 °C/min ramp rate starting at 25 °C. ^b^ In Case 3, Col. A separated propane–heptane, whereas Col. B separated octane–undecane. ^c^ Red font indicates undesirable outcomes compared to other cases.

**Table 4:** Computed ^a^ values of energy consumption for temperature programmed Cases 4–6.

	Case 4	Case 5	Case 6
Stationary Phase Thickness (µm)	Col. B Only: 0.1	Col. A Only: 5.0	Col. A: 5.0 Col. B: 0.1
ΔT_avg_ (°C)	27.5	72.5 ^c^	Col. A: 32.5 Col. B: 27.5
ΔT_max_ (°C)	55	145	Col. A: 65 Col. B: 55
Total Analysis Time (s) ^b^	108	277.8	A: 129/B: 108 Overall: 231
*E_diss_* (J)	41.6	282.0	A: 58.7/B: 41.6 Overall: 100.3
*E_mass_* (J)	38.5	101.5	A: 45.5/B: 38.5 Overall: 84
*E_total_* (J)	80.1	383.5	A: 104.2/B: 80.1 Overall: 184.3

^a^ All cases computed the separation of alkanes C_3_–C_11_ using a 3 m long, 250 µm ID separation column. All cases used a 30 °C/min ramp rate starting at 25 °C. ^b^ Total analysis time is based on the retention times of alkanes in Table 3. ^c^ Red font indicates undesirable outcomes compared to other cases.

## Data Availability

Data sharing not applicable.
